# The causal role of intestinal microbiome in development of pre-eclampsia

**DOI:** 10.1007/s10142-023-01054-8

**Published:** 2023-04-17

**Authors:** Zhihui Xiong, Qingmin Wang, Shuping Pei, Zheng Zhu

**Affiliations:** 1 Obstetrical Department, Tongde Hospital of Zhejiang Province, Hangzhou, 310012 China; 2 Surgical Department, The Second Affiliated Hospital of Zhejiang Chinese Medical University, Xinhua Hospital of Zhejiang Province, Hangzhou, 310005 China

**Keywords:** Placental disease, Mendelian randomization, Intestinal microbiome, Pre-eclampsia, Causality

## Abstract

The correlation of pre-eclampsia (PE) and intestinal microbiome has been widely demonstrated in existing research, whereas their causal relationship has been rarely explored. The causal relationship between intestinal microbiome and PE risk was examined using large-scale genome-wide association studies (GWAS) summary statistics. To be specific, the causal microbial taxa for PE were identified using the two-sample Mendelian randomization (MR) method. The results were verified to be robust through comprehensive sensitive analyses, and the independence of causal relationship was ensured through novel multivariable MR analyses. The possibility of reverse relationships was ruled out through reverse-direction MR analyses. Lastly, the biofunction was explored through enrichment analysis, and a series of validations of PE results in a second GWAS were performed to confirm the results. After correction, four microbial taxa, including *Streptococcus* genus for PE (FDR *q* = 0.085), *Olsenella* genus for PE (FDR *q* = 0.085), *Enterobacteriales* order for PE (FDR *q* = 0.0134), and *Akkermansia* genus for PE (FDR *q* = 0.015), had a causal relationship to diverse joint PE (FDR *q* < 0.15). Moreover, when three different methods were employed on basis of the nominal significance (*P* < 0.05), five suggestive microbial taxa took on significance. The effect of heterogeneity and horizontal pleiotropy was excluded through sensitive analysis, and the possibility of horizontal pleiotropy of BMI was ruled out through multivariable MR analysis. The protective mechanism of the identified taxa against PE was illustrated through GO enrichment analysis and KEGG pathways. A number of microbial taxa had a causal relationship to PE. The result of this study provides more insights into intestinal microbiome in the pathology of PE.

**Supplementary Information:**

The online version contains supplementary material available at 10.1007/s10142-023-01054-8.

## Introduction

Pre-eclampsia (PE) occurs during pregnancy, which has been confirmed to be associated with multiple system diseases. PE is recognized as one of the most common pregnancy complications (El-Sayed 2017), and it can complicate 2–8% of global pregnancies (ACOG Practice Bulletin No 2019). PE results in several adverse consequences (e.g., kidney failure, acute pulmonary edema, fetal growth restriction, fetal distress, placental abruption, and stillbirth) (Soh et al. 2015; Gottardi et al. 2021). Existing research has suggested that the pathogenesis of PE may be correlated with oxidative stress, abnormal lipid metabolism, endothelial cell activation damage and so forth, whereas the specific pathogenesis has not been clarified (Ding et al. 2014). Around the world, nearly 50,000 pregnant women die every year from PE or eclampsia (Filipek and Jurewicz 2018). For instance, 15% of maternal deaths in the UK are caused by PE and eclampsia, of which 2/3 are associated with PE, as indicated by existing research (Filipek and Jurewicz 2018). In 2014, Abalos et al. (Abalos et al. 2014) suggested that the incidence rate of PE, eclampsia, and chronic hypertension in pregnancy reached 2.16%, 0.29%, and 0.28%, respectively. Accordingly, it is imperative to place sufficient attention to PE.

Intestinal microbiome refers to a complex microbial community living in the digestive tract, significantly affecting metabolism, immunity, and nutrient absorption of the body (Huber et al. 2007; Zhang et al. 2023). The imbalanced intestinal microbiome composition or function has a close correlation with numerous diseases (e.g., spirit, immune system, blood system, cardiovascular system, endocrine system, psychology, and nervous system) (Zhou et al. 2020; Chen et al. 2021, 2022). During normal pregnancy, pregnant women undergo several physiological changes (e.g., immune, endocrine, and metabolic adaptation) to facilitate the growth of the fetal placenta. Moreover, the intestinal microbiome and its metabolism will undergo changes regarding host physiology and immune adaptation (Zhang et al. 2015). The imbalanced intestinal microbiome in pregnant women plays a certain role in the occurrence of pregnancy complications (e.g., PE) by affecting the maternal immune and metabolic functions (Edwards et al. 2017; Gomez-Arango et al. 2016). In 2016, the 16rDNA sequencing of stool samples from 26 PE patients and healthy pregnant women in the third trimester were compared by some scholars, and the result indicated that the composition and structure of intestinal microorganisms in PE patients change significantly, and *Clostridium* and *Bulleidia* exhibit significantly increased abundance (Liu et al. 2017). Subsequently, relevant research (Lv et al. 2019) has further revealed that the intestinal microbiome of patients with early-onset PE also changes significantly, *Fusobacterium*, *Blautia*, and other bacteria exhibit an enhanced abundance, while *Akkermansia* and *Faecalibacterium* exhibit a decreased abundance. As revealed by the above research, the intestinal microbiome of patients with PE is notably imbalanced, which is associated with the occurrence and development of the disease. However, extensive studies have reported different PE characteristic bacterial spectra, there is no homogeneity, and most of them are case–control studies; the number of cases has been limited. Besides it is difficult to realize the causal evidence presented by RCT or large cohort studies.

Mendelian randomization (MR) is capable of identifying the causal relationship between risk factors and diseases with genetic variation as an instrumental variable (Vandebergh et al. 2022). Since genetic variation has been determined at the time of conception, it is not susceptible to other factors or confounding. Moreover, MR research meets the causal timing, which takes on significance to causal inference and lays a basis for causal relationship (Vandebergh et al. 2022). Accordingly, based on the public data regarding the whole genome, two-way MR analysis was performed on two samples to investigate the correlations between different intestinal microbiome and PE risk, and the robustness of the results was verified through comprehensive sensitivity analysis in accordance with the specific effect of different intestinal microbiomes on PE.

## Material and methods

### Research design

A two-sample two-way MR design method was employed to verify whether there is a causal relationship between intestinal microbiome and PE.

### Data source

The datasets applied can be publicly accessible: GWAS summary statistics in terms of intestinal microbiome were the exposure that originated from the MiBioGen consortium (http://www.mibiogen.org/) (Kurilshikov et al. 2021). Gut microbiota involved in this study were divided into 211 taxa (131 genera, 35 families, 20 orders, 16 classes, and 9 phyla) and a total of 5,717,754 SNPs genotyped by 16S fecal microbiome were analyzed on 18,340 individuals (24 cohorts). The correlation between the gut microbiome and autosomal human genetic variants was explored using a multi-ethnic large-scale GWAS recruiting 20 population-based cohorts from European, African American, American Hispanic/Latin, East Asian, and Middle Eastern. A large PE GWAS provided the effect estimates of the SNPs regarding PE risk, comprising 264,887 controls of European ancestry and 2355 cases (Sakaue et al. 2021).

### Selection of instrumental variable

First, important SNPs were selected for the respective intestinal microbiota using a cut-off value of *P* < 1E-5. Second, the clump program in PLINK software (Purcell et al. 2007) was adopted to exclude the dependent instrumental variable of *r*^2^ < 0.001 (clumping window size = 10,000 kb), which was obtained using the 1000 Genome Projects reference panel in Europe. If instrumental variable (IV) is missing from the result dataset, we will add an agent with *r*^2^ > 0.8. Subsequently, four to 25 separate IVs were retained for further analysis for all intestinal microflora. Lastly, to quantify the intensity of IV, we calculated the variance interpretation ratio (PVE) and *F* statistic of each microbial group IV. Table S1 lists SNPs applied in the analysis.

### Mendelian randomization

In this study, inverse variance weighted (IVW) method was primarily employed, in which the existence of the intercept term was not considered in regression, and the reciprocal of outcome variance served as the weight for fitting. To be specific, the IVW fixed effect model was largely adopted when there was no potential level pleiotropic heterogeneity (Burgess et al. 2013). Heterogeneity was detected using Cochran’s *Q* statistics. If Cochran’s *Q* statistics test achieved statistical significance, the analysis results exhibit significant heterogeneity. The random effect model will be employed if there is heterogeneity. Second, the above conclusions were further supplemented using weighted medium, MR Egger, MR PRESSO, and other methods. The weighted median method is the median of the weighted empirical density function of the ratio estimates. The causality can be evaluated if at least 50% of the information in the analysis originates from effective instrumental s (Bowden et al. 2016). MR Egger intercept detection was conducted to determine whether there is level pleiotropy in MR analysis. If there is an intercept term in MR Egger intercept analysis (*P* intercept < 0.05), the study will have significant horizontal pleiotropy, and the slope of MR Egger slope will be a relatively accurate effect estimate, since this method reduces the hypothesis errors due to pleiotropy (Bowden et al. 2015). MR-PRESSO first calculates the IVW result after removing the SNPs for the respective SNP, and then calculates the residual square sum of the SNPs’ effect and the IVW result. Lastly, we add the residual square sum (distance) calculated by using the respective SNP. The larger this value, the more significant the horizontal pleiotropy will be (Verbanck et al. 2018). Besides, the global test was performed in MR-PRESSO to detect outliers. If there are outliers, they should be eliminated and reanalyzed.

Besides the test of the reliability and stability of the results using the above three methods (weighted medium, MR Egger, and MR-PRESSO), sensitivity analysis was conducted using the leave one out method. To be specific, for the intestinal microbiome whose *P* value was less than 0.05 in the IVW method and which has passed the level pleiotropy and heterogeneity tests, the respective related SNP was removed, and the combined effect of the remaining SNPs was calculated to evaluate the effect of the respective SNP on the gut microbiota.

False positive in multiple tests was controlled using the FDR method. FDR *q* < 0.15 was considered significant, while *P* < 0.05 indicated a potential causal association. R 4.1.0 version was adopted for all statistical analysis, and Mendelian randomization, MR-PRESSO, and other R packages were employed (Vandebergh et al. 2022).

Furthermore, considering the critical impact of BMI on the progress from gut microbiota to PE onset, we used a multivariable MR analysis to rule out the potential pleiotropy. Notably, SNPs related to both causal gut microbiota and BMI were utilized as IVs for multivariable MR analysis. Then, the multivariable MR analysis was performed to estimate the causal effect of gut microbes on PE after adjusting for BMI (Yengo et al. 2018). Additionally, a two-sample MR validation analysis was performed with PE as the outcome using GWAS summary data of European individuals from the finn-b-O15_PREECLAMPS consortium, including 3556 cases vs. 114,735 controls.

### GO and KEGG enrichment analysis

To further explore the biological role of gut microbial taxa on the development of PE, we performed GO and KEGG enrichment analyses based on lead SNPs for all identified gut microbial taxa. All analyses were conducted using R software (version 4.1.1) combined with the “Mendelian Randomization” package (version 0.4.3) specifically used in the MR and multivariable MR analyses. MR-PRESSO was conducted with the help of the “MR-PRESSO” package; GO and KEGG enrichment were performed with the “FUMA” website tool to map the instrumental variable to the nearby gene. After that, the pathway and tissue enrichment analyses were carried out also for the nearby gene.

## Results

### Causal effect of intestinal microbiome on PE

**Fig. 1:**
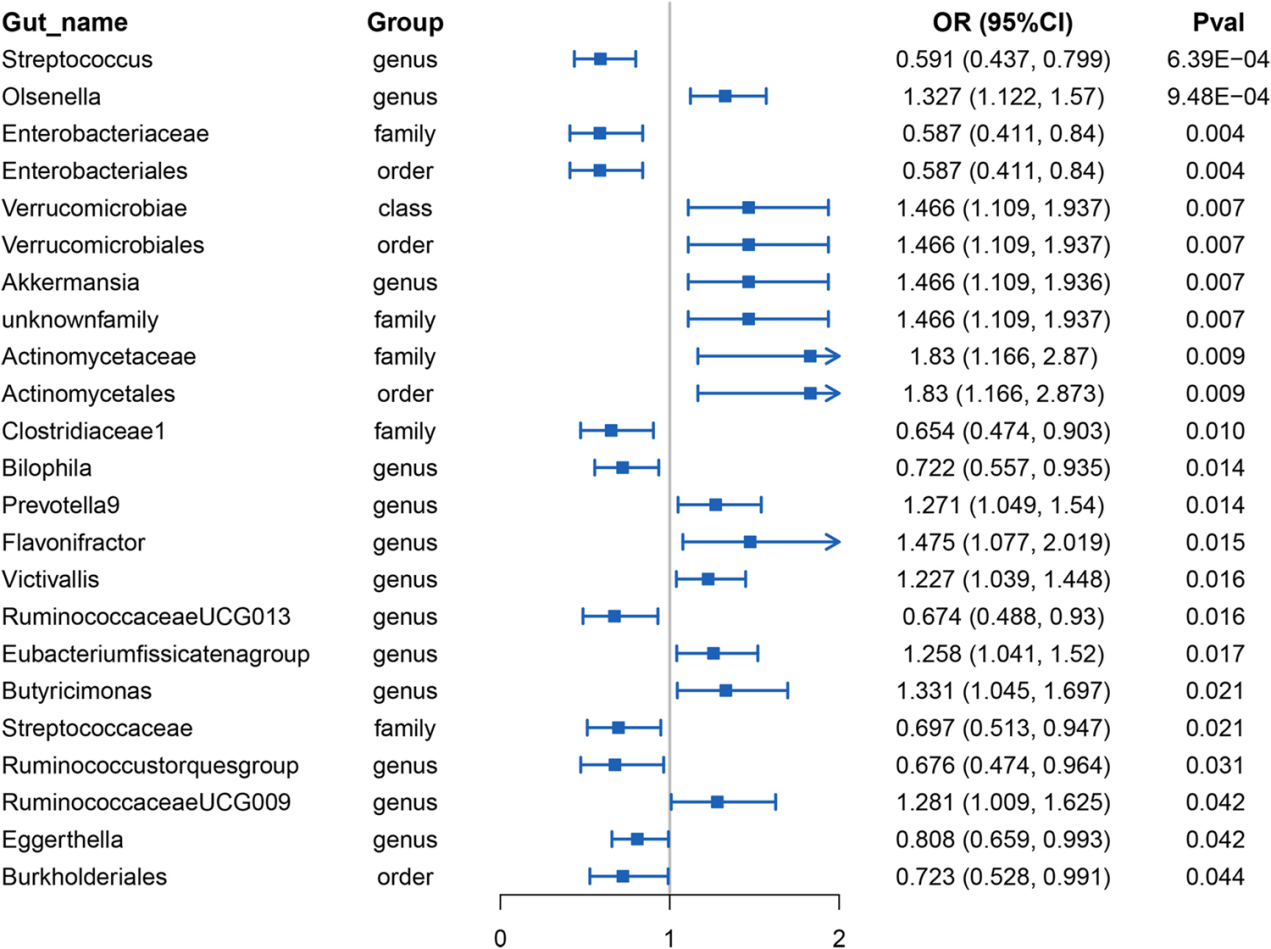
Forest plot for causal association between 23 unique causal/suggestive microbial taxa with PE. OR, odds ratio; CI, confidence interval; PE, pre-eclampsia; IVW, inverse-variance weighted, MVMR, multivariable variable Mendelian randomization

Several significant results regarding the causal relationship between PE risk and intestinal microbiome were identified through IVW methods. A total of 23 microbial taxa were found at the nominal significance level (*P* < 0.05), which involved 3 orders, 12 genera, and 5 families. Figure 1 lists causal features that are potentially significant. Twenty-three microbial taxa took on significance in terms of PE. However, after FDR correction (FDR *q* < 0.15), there were still four microbial taxa in terms of PE (Table 1).

**Table 1:** Forest plot for causal association between 4 unique causal microbial taxa with PE. *OR*, odds ratio; *CI*, confidence interval; *PE*, pre-eclampsia; *IVW*, inverse-variance weighted, *MVMR*, multivariable variable Mendelian randomization. For PE, four taxa are causal (i.e., *Streptococcus* genus, *Olsenella* genus, *Enterobacteriales* order, and *Akkermansia* genus). The bold parts of the *P*-value are significant

Gut name	Group	Methods	OR (95% CI)	Pval	Heterogeneity test (P)	MR-PRESSO global test (P)
*Streptococcus*	Genus	IVW	0.591 (0.437, 0.799)	**6.39E-04**	5.836 (0.970)	6.648 (0.974)
		Weighted mode	0.686 (0.413, 1.139)	0.145		
		Weighted median	0.64 (0.429, 0.954)	**0.028**		
		MR Egger (slope)	0.625 (0.201, 1.945)	0.417		
		MR Egger (intercept)	− 0.004 (− 0.092, 0.083)	0.920		
		MR-PRESSO	0.591 (0.486, 0.718)	**1.15E-04**		
		MVMR (BMI adjusted)	0.751 (0.323, 1.749)	0.507		
*Olsenella*	Genus	IVW	1.327 (1.122, 1.57)	**9.48E-04**	3.285 (0.952)	3.988 (0.962)
		Weighted mode	1.404 (1.002, 1.968)	0.049		
		Weighted median	1.379 (1.102, 1.727)	**0.005**		
		MR Egger (slope)	1.401 (0.758, 2.589)	0.282		
		MR Egger (intercept)	− 0.007 (− 0.086, 0.071)	0.857		
		MR-PRESSO	1.327 (1.199, 1.469)	**3.95E-04**		
		MVMR (BMI adjusted)	1.146 (0.807, 1.629)	0.447		
*Enterobacteriales*	Order	IVW	0.587 (0.411, 0.84)	**0.004**	6.056 (0.641)	7.653 (0.680)
		Weighted mode	0.678 (0.389, 1.183)	0.171		
		Weighted median	0.617 (0.381, 0.998)	**0.049**		
		MR Egger (slope)	0.555 (0.079, 3.9)	0.507		
		MR Egger (intercept)	0.005 (− 0.148, 0.158)	0.948		
		MR-PRESSO	0.587 (0.43, 0.802)	**0.010**		
		MVMR (BMI adjusted)	0.262 (0.04, 1.723)	0.164		
*Akkermansia*	Genus	IVW	1.466 (1.109, 1.936)	**0.007**	5.016 (0.890)	6.106 (0.885)
		Weighted mode	1.823 (0.991, 3.355)	0.054		
		Weighted median	1.604 (1.112, 2.312)	**0.011**		
		MR Egger (slope)	2.777 (1.07, 7.208)	**0.036**		
		MR Egger (intercept)	− 0.053 (− 0.129, 0.023)	0.170		
		MR-PRESSO	1.466 (1.203, 1.786)	**0.003**		
		MVMR (BMI adjusted)	0.793 (0.411, 1.532)	0.490		

**Fig. 2:**
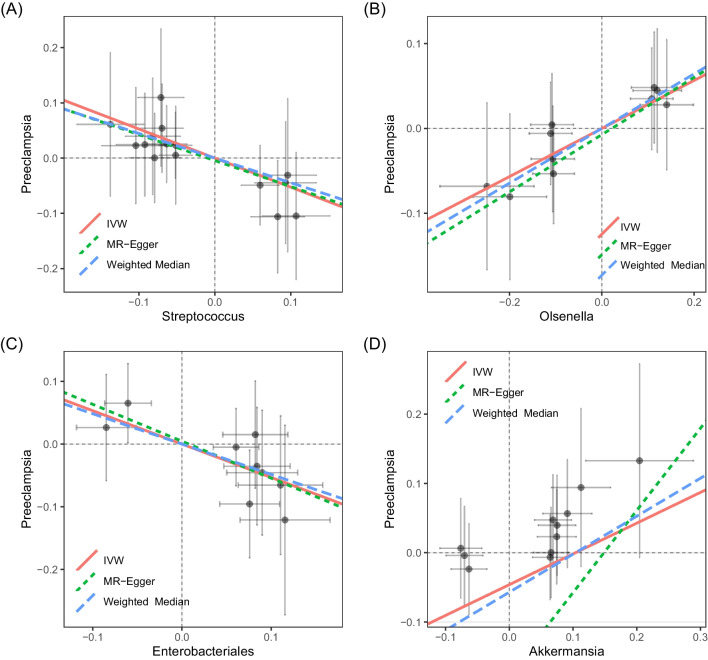
Causal association between causal microbial taxa with PE. In **A**–**D**, red lines represent estimations with IVW method and green dotted lines represent estimations with weighted median method. **A** Scatter plot for causal effect of *Streptococcus* genus on PE. **B** Scatter plot for causal effect of *Olsenella* genus on PE. **C** Scatter plot for causal effect of *Enterobacteriales* order on PE. **D** Scatter plot for causal effect of *Akkermansia* genus on PE. PE, pre-eclampsia; IVW, inverse-variance weighted

To be specific, *Streptococcus* genus had a negative relationship to PE risk (FDR *q* = 0.085). The same results (*P*_IVW_ = 6.39E-04, *P*_Weighted-Median_ = 0.028, *P*_Mr-Presso_ = 1.15E-04) were achieved with the use of three different methods. The increased *Olsenella* genus led to the increased risk of PE (*P*_IVW_ = 9.48E-04, FDR *q* = 0.085), the same as MR-PRESSO (*P*_Mr-Presso_ = 3.95E-04) and weighted-median (*P*_Weighted-Median_ = 0.005). Besides, *Enterobacteriales* order’s causal effect on PE took on significance on the basis of three different methods (*P*_IVW_ = 0.004, FDR *q* = 0.013, *P*_Weighted-Median_ = 0.049, *P*_Mr-Presso_ = 0.010). Moreover, *Akkermansia* genus coded to be ID 4037 took on 0.05 at the nominal significance level on the basis of three different methods (*P*_IVW_ = 0.007, *P*_Weighted Median_ = 0.011, *P*_Mr-Presso_ = 0.003), and it led to the increased PE risk (Table 1). The causal relationships between intestinal microbiome for PE were verified to be robust in accordance with the sensitive results (Table S2). Nevertheless, intercept of MR Egger showed no potential horizontal pleiotropy in terms of the causal effect on PE (*P*_Egger-intercept_ = 0.920 for *Streptococcus* genus, *P*_Egger-intercept_ = 0.857 for *Olsenella* genus, *P*_Egger-intercept_ = 0.948 for *Enterobacteriales* order, *P*_Egger-intercept_ = 0.170 for *Akkermansia* genus). Furthermore, the possibility of horizontal pleiotropy (*P* global test = 0.974 in terms of *Streptococcus* genus, *P* global test = 0.962 in terms of *Olsenella* genus, *P* global test = 0.680 in terms of *Enterobacteriales* order, and *P* global test = 0.885 in terms of *Akkermansia* genus) was ruled out through global test of MR-PRESSO. Next, funnel and scatter plots were generated for causal and suggestive microbial taxa, and the results indicated that our estimation was not significantly affected by any potential outlier (Fig. 2 and S1). No large-effect-size SNP biased the estimation of this study (Fig. S2) as indicated by the LOOCV results.

The validation analysis confirmed our findings for the association between *Olsenella* and PE showing similar and consistent effect estimates but with a loss of statistical significance (quantified by *P* values in Fig. S3). Thus, genetically predicted *Streptococcus* was inversely associated with PE (OR IVW = 1.170, 95% CI: 0.911; 1.503, *P* = 0.219) (Fig. S3). The same applies to the relationship between *Enterobacteriales* and PE (OR IVW = 0.640, 95% CI: 0.459; 0.894, *P* = 0.009). Again, no associations could be observed between *Akkermansia* and PE (Fig. S3).

### Multivariable MR analysis

Multivariable MR analysis was performed to adjust BMI, such that if horizontal pleiotropy changed the results of this study was examined in depth. After the adjustment in terms of BMI, the possibility of a potentially pleiotropic effect was ruled out (Table 2). For PE, the independent causal effect of *Ruminococcaceae* genus was observed, with an odds ratio of 0.532 (95% CI: 0.352–0.872, *P* mvmr = 0.012) for the *Ruminococcaceae* genus on PE, where similar results were achieved in terms of the *Butyricimonas* genus on PE (OR = 1.674, 95% CI: 1.140–2.458, *P* mvmr = 0.009) and *Streptococcaceae* family (OR = 0.529, 95% CI: 0.316–0.887, *P* mvmr = 0.016). Nevertheless, after adjustment, the multivariable results in terms of suggestive microbial taxa were not significant, thus revealing that BMI may exert a pleiotropic effect on the development of PE and gut microbial taxa (Table 2).

**Table 2:** The possibility of potentially pleiotropic effect after adjusting for BMI. *OR*, odds ratio; *CI*, confidence interval; *PE*, pre-eclampsia; *MVMR*, multivariable variable Mendelian randomization. For PE, three taxa are causal (i.e., *Ruminococcaceae* genus, *Butyricimonas* genus, and *Streptococcaceae* family)

Gut microbiota	Variables	BETA	SE	LOW	UP	OR	*P*
genus.Streptococcus.id.1853	Gut microbiota BMI adjusted	− 0.286	0.431	0.751	0.323	1.749	0.507
genus.Olsenella.id.822	Gut microbiota BMI adjusted	0.136	0.179	1.146	0.807	1.629	0.447
family.Enterobacteriaceae.id.3469	Gut microbiota BMI adjusted	− 1.338	0.960	0.262	0.040	1.723	0.164
order.Enterobacteriales.id.3468	Gut microbiota BMI adjusted	− 1.338	0.960	0.262	0.040	1.723	0.164
class.Verrucomicrobiae.id.4029	Gut microbiota BMI adjusted	− 0.232	0.336	0.793	0.411	1.531	0.490
order.Verrucomicrobiales.id.4030	Gut microbiota BMI adjusted	− 0.232	0.336	0.793	0.411	1.531	0.490
genus.Akkermansia.id.4037	Gut microbiota BMI adjusted	− 0.232	0.336	0.793	0.411	1.532	0.490
family.Verrucomicrobiaceae.id.4036	Gut microbiota BMI adjusted	− 0.232	0.336	0.793	0.411	1.531	0.490
family.Actinomycetaceae.id.421	Gut microbiota BMI adjusted	− 0.720	NaN	0.487	#VALUE!	#VALUE!	NaN
order.Actinomycetales.id.420	Gut microbiota BMI adjusted	− 0.723	NaN	0.485	#VALUE!	#VALUE!	NaN
family.Clostridiaceae1.id.1869	Gut microbiota BMI adjusted	− 0.231	0.325	0.793	0.420	1.499	0.476
genus.Bilophila.id.3170	Gut microbiota BMI adjusted	− 0.065	0.287	0.937	0.534	1.644	0.820
genus.Prevotella9.id.11183	Gut microbiota BMI adjusted	0.342	0.203	1.408	0.946	2.096	0.092
genus.Flavonifractor.id.2059	Gut microbiota BMI adjusted	− 0.196	0.290	0.822	0.465	1.452	0.499
genus.unknowngenus.id.959	Gut microbiota BMI adjusted	− 0.045	0.180	0.956	0.671	1.361	0.802
genus.RuminococcaceaeUCG013.id.11370	Gut microbiota BMI adjusted	− 0.631	0.252	0.532	0.325	0.872	0.012
genus..Eubacteriumfissicatenagroup.id.14373	Gut microbiota BMI adjusted	− 0.002	0.192	0.998	0.685	1.453	0.991
genus.Butyricimonas.id.945	Gut microbiota BMI adjusted	0.515	0.196	1.674	1.140	2.458	0.009
family.Streptococcaceae.id.1850	Gut microbiota BMI adjusted	− 0.637	0.264	0.529	0.316	0.887	0.016
genus..Ruminococcustorquesgroup.id.14377	Gut microbiota BMI adjusted	0.458	0.292	1.581	0.893	2.801	0.116
genus.RuminococcaceaeUCG009.id.11366	Gut microbiota BMI adjusted	0.072	0.248	1.074	0.661	1.747	0.772
genus.Eggerthella.id.819	Gut microbiota BMI adjusted	− 0.368	NaN	0.692	#VALUE!	#VALUE!	NaN
order.Burkholderiales.id.2874	Gut microbiota BMI adjusted	− 2.973	2.679	0.051	0.000	9.760	0.267

### Reverse-direction MR analyses

Lastly, PE did not have a causal effect on 23 gut microbial taxa (Table S3), as indicated by the result of the reverse MR analysis. However, a potential significant causal effect was identified (*P*_IVW_ = 0.049) only for the correlation between any PE with the *Akkermansia* genus, and no significant causal associations were detected with other methods

### GO and KEGG enrichment analysis

**Fig. 3:**
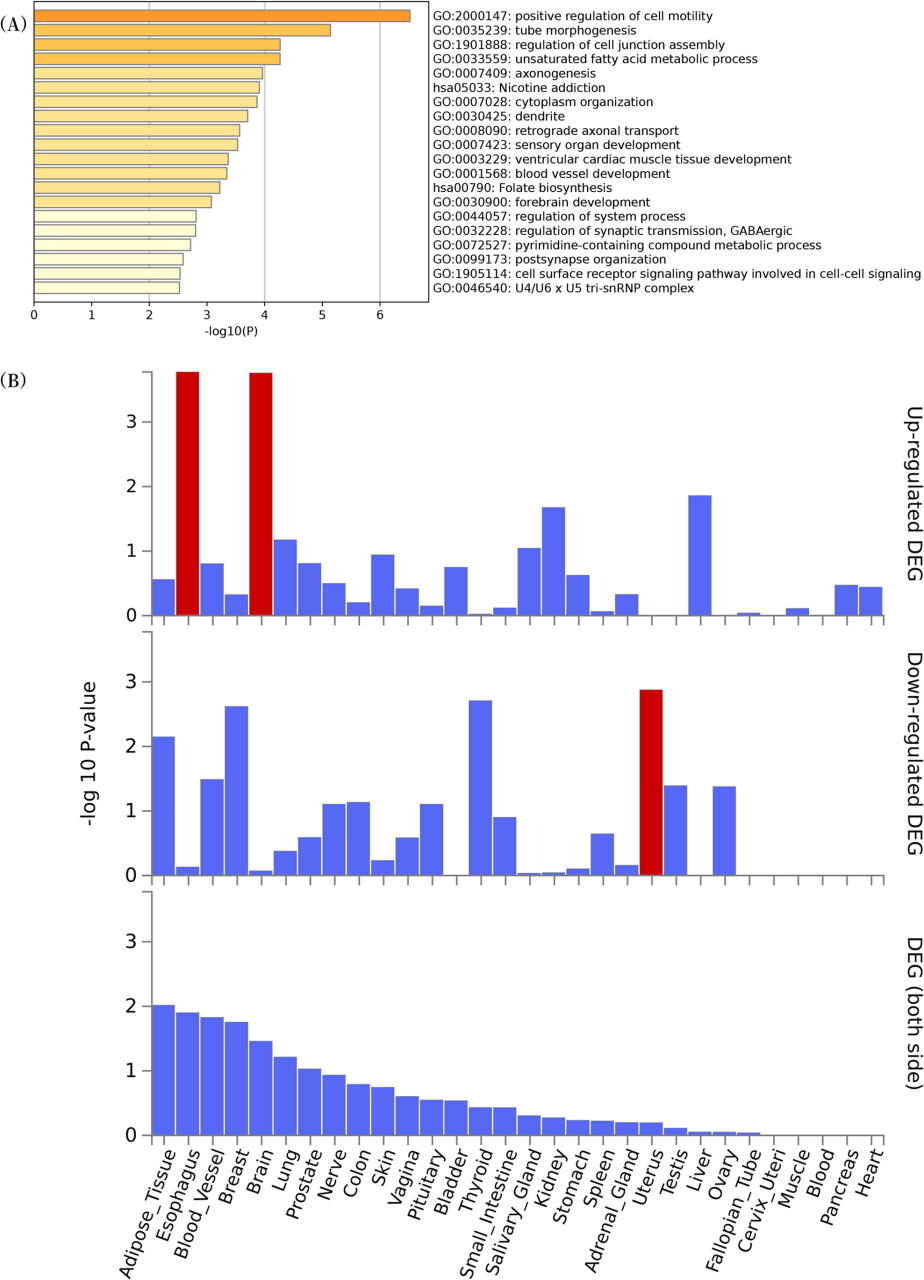
Functional analyses for the nearby genes mapped by gut-related IVs. **A** GO enrichment analyses indicated that the nearby genes were significantly related to the function of “positive regulation of cell motility”, “tube morphogenesis”, and “unsaturated fatty acid metabolic process”. **B** KEGG pathway analyses indicated that nicotine addiction and folate biosynthesis were enriched. IVs, instrumental variables

The significant enrichment of several crucial regulation pathways was indicated by the result of the GO term enrichment analysis regarding microbial taxa on PE. In terms of PE, 19 GO terms (e.g., positive regulation of cell motility, tube morphogenesis, and unsaturated fatty acid metabolic process) were observed to be involved in PE (Fig. 3A). Moreover, two KEGG pathways (e.g., nicotine addiction and folate biosynthesis) played a certain role in PE (Fig. 3B).

## Discussion

To the best of our knowledge, this has been the initial comprehensive and in-depth research on the causal relationships between intestinal microbiome and PE risk based on publicly available genetic databases. On the basis of 18,340 individuals (24 cohorts), comprehensive MR analyses were conducted in terms of 211 taxa for revealing the potential role played by the intestinal microbiome in the development of PE. Multiple gut microbial taxa, including four causal microbial taxa (i.e., *Streptococcus* genus, *Olsenella* genus, *Enterobacteriales* order, and *Akkermansia* genus) and 19 suggestive microbial taxa, take on a critical significance to the development of PE, as revealed by the findings.

The experimental result indicated a decreased number of *Streptococcus* species in the PE group, thus suggesting that *Streptococcus* bacteria may be correlated with the occurrence of PE (Chang et al. 2020). Other research (Mulla et al. 2013) has suggested that *Streptococcus* may be associated with tumor necrosis factor (TNF)-alpha, interleukin-6 (IL-6), and other cytokines. *Streptococcus* induced TNF-α (Zaga et al. 2004; Kwak et al. 2000; Rosati et al. 1998; Hunolstein et al. 1997) and IL-6 (Zaga et al. 2004; Kwak et al. 2000; Rosati et al. 1998). TNF-α is related to PE and may be a pathogenic factor in early eclampsia (Schipper et al. 2005; Beckmann et al. 2004, 1997; Visser et al. 2002, 1994; Chen et al. 1996). IL-6 may play a similar role (Conrad et al. 1998; Greer et al. 1994). TNF-α is capable of desensitizing its receptor, thus reducing its receptor signal when repeatedly stimulated (Wallach et al. 1988; Gaeta et al. 2000; Karmann et al. 1996). In addition, TNF exposure may downregulate IL-6 expression (Barnes et al. 1998). A possible reason for the lower incidence rate of PE in our population is that the TNF-α receptor of *Streptococcus*-colonized women is relatively desensitized due to long-term exposure to TNF-α. The results of this study indicated that *Streptococcus* protected PE, thus suggesting that *Streptococcus* may play a certain role in the possible mechanism of regulating PE progress by affecting TNF-α. The comprehensive MR analysis of this study indicated that the *Enterobacteriales* order had a protective effect on PE risk, whereas the effect of *Enterobacteriales* order in the intestinal microbiota on PE development remained unclear. A clinical experiment indicated that the increase in the number of *Enterobacteriales* order leads to the imbalance of gut microbiota, increases the risk of harmful bacteria infection, and activates the inflammatory signaling pathways (Huang et al. 2018), which is not consistent with the results of this study. Moreover, *Akkermansia* can facilitate PE, whereas this relationship was not supported by the MR results. As revealed by this study, the above inconsistency arises from inevitable confounding factors in population research, which have been frequently identified in microbiome research. In addition, *Olsenella* has been confirmed to play a certain role in unsaturated fatty acid biosynthesis (Zhan et al. 2021). The levels of n-6 fatty acids and n-3 fatty acids in the early pregnancy of PE were decreased, the levels of n-3 fatty acids showed a more significant decrease than those of n-6 fatty acids, and the ratio of n-6/n-3 was increased, as indicated by the experimental result (Wadhwani et al. 2016). The increase of n-6/n-3 ratio is related to oxidative stress reaction, and has the effects of promoting vasoconstriction, oxidative stress, inflammation, and coagulation, which are considered the critical reasons for unsaturated fatty acid disorder to increase the occurrence of PE.

Some suggestive intestinal microbiotas have also been found in MR analysis, and part of these microbiotas have been confirmed by existing observational research. *Bilophila* was also the major variance in PE microbiomes. It is capable of promoting higher inflammation through the production of hydrogen sulfide (Silva et al. 2008), bile acid dysmetabolism, and intestinal barrier dysfunction (Devkota et al. 2012; Natividad et al. 2018). Moreover, *Bilophila* in higher amounts is capable of releasing LPS and IL-6 (Hunter and Jones 2015 May), which is consistent with the observation; i.e., the abundance of intestinal *Bilophila* is positively correlated with the prenatal plasma IL-6 level. As indicated by the major results of existing research, women with PE achieved higher plasma levels of the proinflammatory cytokine TNF-α, as well as a higher relative abundance of *Prevotella bivia* in the vaginal microbiota (Hung et al. 2004). The above research suggests that PE is accompanied by a systemic inflammatory response (Ishida et al. 2019), and the ischemic placental injury under this condition is related to an increased release of TNF-α in the maternal bloodstream (Lau et al. 2013). When a multiplex assay was performed for eight different cytokines, TNF-α was the only increased inflammatory biomarker in the plasma of this study’s PE cases. It is noteworthy that the preponderance of *Prevotella oralis* in the oral microbiome is previously related to older women’s blood pressure outcomes. On that basis, the *Prevotella* genus may affect the origination and development of hypertension, as well as in pregnancy’s hypertensive disorders. This also supports the validity of the results of this study. It is interesting that an unknown genus coded to be 959 takes on significance in PE at a nominal significance level, whereas its specific information and biological functions remain unclear. Thus, in-depth research should be conducted on this unknown genus.

In addition, the result of GO enrichment analysis indicated that numerous GO biological processes are critical to the correlation between intestinal microbiome and PE, as demonstrated by existing research. For instance, positive regulation of cell mobility and tube morphogenesis are related to the development and progression of PE. In mammals, the critical event in the development of normal placenta is the establishment of an effective maternal circulation, which is associated with the differentiation of trophoblastic cells into invasive extravillous trophoblastic cells and multinuclear trophoblastic cells (Moser et al. 2018). Any abnormality of trophoblast cell differentiation may cause severe pregnancy disorder (e.g., PE) (Xu et al. 2019). Most research has suggested that PE is caused by trophoblast cells invading the surface layer of the uterine wall (Su et al. 2021; Zhang et al. 2021; Ridder et al. 2019). Accordingly, the failure of incomplete spiral artery remodeling will result in the abnormal imbalance of circulating angiogenic factors and further trigger maternal endothelial dysfunction, thus leading to PE (Stepan et al. 2020). As revealed by some evidence, unsaturated fatty acids play multiple roles in membrane structure, lipid metabolism, blood coagulation, and blood pressure, and they show a correlation with cardiovascular diseases. The metabolic process of unsaturated fatty acids was associated with PE, as indicated by the result of our enrichment analysis. Existing literature has suggested that the peroxidation of unsaturated fatty acids may jeopardize atherosclerotic arteries by releasing toxic endoperoxide and arachidonic acid metabolizing thromboxane (i.e., platelet aggregation agent) (Choi et al. 2020). Accordingly, some clinical experiments suggested an accumulation of unsaturated fatty acids in the placental tissues of PE (Devarshi et al. 2019). Moreover, unsaturated fatty acids may significantly regulate fat metabolism of placenta and fetus. Existing research has suggested that arachidonic acid (AA) can upregulate inflammatory response, while docosapentaenoic acid (EPA) and docosahexaenoic acid (DHA) can downregulate inflammatory response (Rani et al. 2016). We speculate that intestinal microbiome may affect PE by affecting unsaturated fatty acids. Furthermore, the result of KEGG pathway analyses indicated the enrichment of the signaling by nicotine addiction and folate biosynthesis. Nicotine addiction has a negative impact on maternal health; the risks of smoking in pregnancy should include not only fetal risks but maternal risks (including pre-eclampsia) as well (Roelands et al. 2009). Interestingly, according to a study, women who smoke cigarettes throughout pregnancy are at a 33% reduced risk of developing this disorder (Bainbridge et al. 2005). In addition, folate is an essential micronutrient during pregnancy, deficient folate status in association with alteration in expression of enzymes involved in folate metabolism might be associated with pregnancy complications such as pre-eclampsia and NTDs (Mohanraj et al. 2019). We surmise that nicotine addiction and folate biosynthesis can also cause changes in gastrointestinal microbiome; the exact mechanisms through gastrointestinal microbiome that influence the risk of pre-eclampsia need to be studied ulteriorly.

Notably, changes in intestinal microbiome that are mediated by obesity are capable of causing local and systemic inflammation of low grades, thus causing PE attacks. The pleiotropic effect of BMI on the development of pathogenic intestinal microbiota and PE was excluded through the multivariate MR analysis. The statistically insignificant results of intestinal microbiota may arise from the limited statistical capacity or the potential pleiotropic effect of BMI.

In brief, the intestinal microbiota related to PE were comprehensively screened in this study. The results of this study also indicated the protective role of a wide variety of intestinal microbiota in the development of PE, thus guiding PE prevention and treatment in clinical practice. Nevertheless, this study has some limitations. To be specific, nearly 100 genetic variations related to intestinal microbiome were identified using GWAS, and the effect of a single genetic variation on intestinal microbiome was extremely weak (OR values of disease susceptibility loci identified by most GWAS are < 2.0), which can only account for a small part of the heritability of intestinal microbiome (Roelands et al. 2009). Furthermore, BMI was corrected to further evaluate the risk of the intestinal microbiome to PE, and BMI serves as an indirect indicator. Besides, fat and muscle accumulation cannot be distinguished, and fat distribution cannot be effectively identified (Indiani et al. 2018). Third, although most participants in the GWAS meta-analysis for gut microbiota data were European descendants, we still consider that our data may have interference from population stratification, so it is worth mentioning that the results of this study may not be entirely applicable to non-European subjects. Future MR studies on the causal association between gut microbiota and PE are recommended to embrace data from diverse European and non-European populations and ensure the generalizability and applicability of the outcomes. Fourth, since there are insufficient personal data, in-depth population stratification research (e.g., gender) cannot be conducted, and possible differences in different populations can be investigated. Lastly, we additionally included large genetic consortia for PE for validation. Notably, these GWAS did not adjust for gender; the cases were females, but nevertheless the non-cases included males, which could confound the MR estimates likely away from the null.

## Supplementary Information

Below is the link to the electronic supplementary material.

Supplementary file1 (PDF 10 KB)

Supplementary file2 (PDF 5 KB)

Supplementary file3 (PDF 128 KB)

Supplementary file4 (XLSX 12 KB)

Supplementary file5 (XLSX 119 KB)

Supplementary file6 (XLSX 13 KB)

## Data Availability

The raw data supporting the conclusions of this article will be made available by the authors, without undue reservation.
