# Supplementary figures and images for: The causal role of intestinal microbiome in development of pre-eclampsia
Source: Funct Integr Genomics. 2023 Apr 17;23(2):127. doi: 10.1007/s10142-023-01054-8 (PMC10110674; doi:10.1007/s10142-023-01054-8)

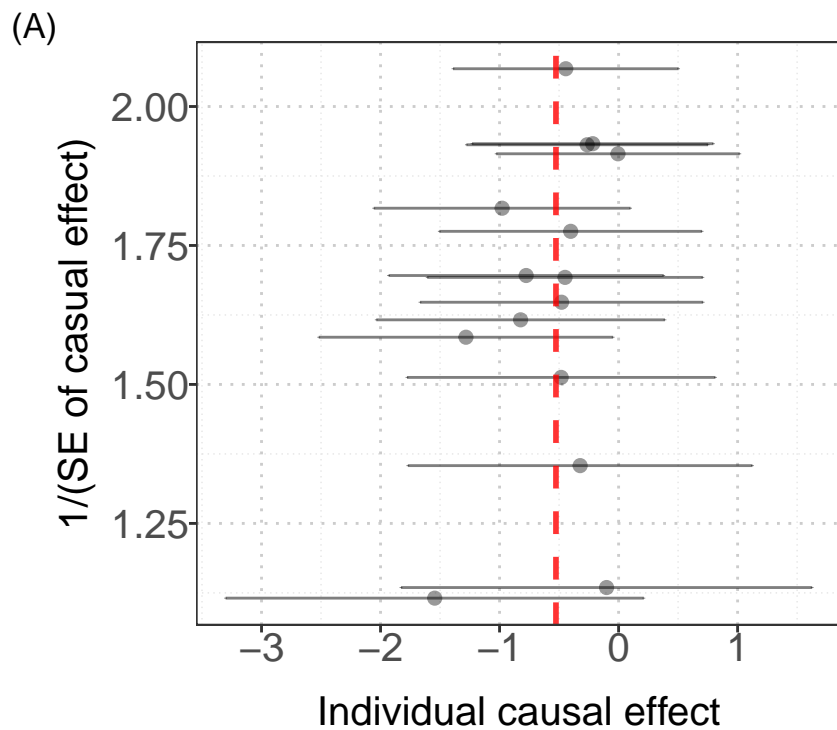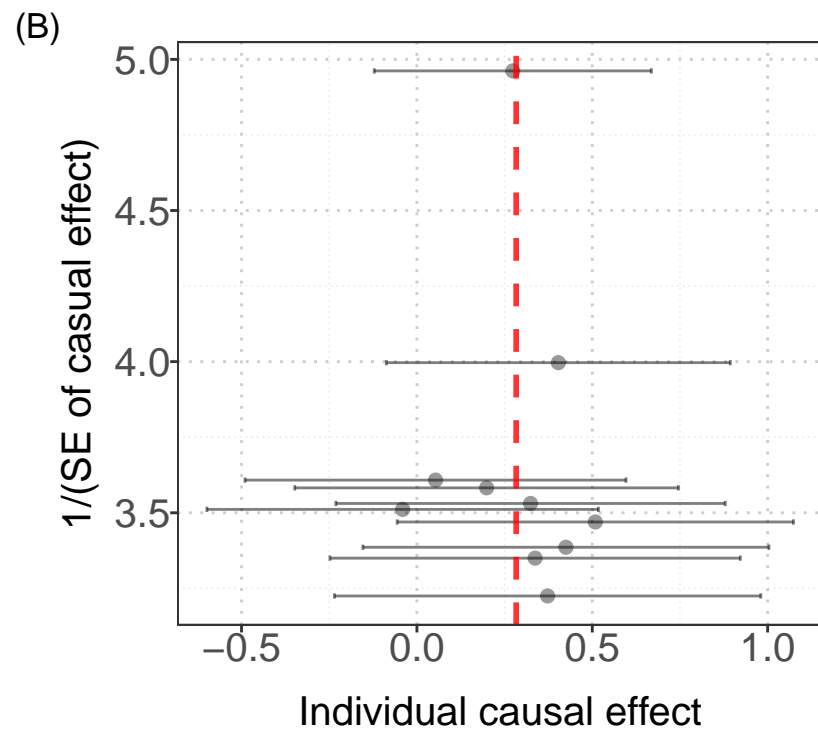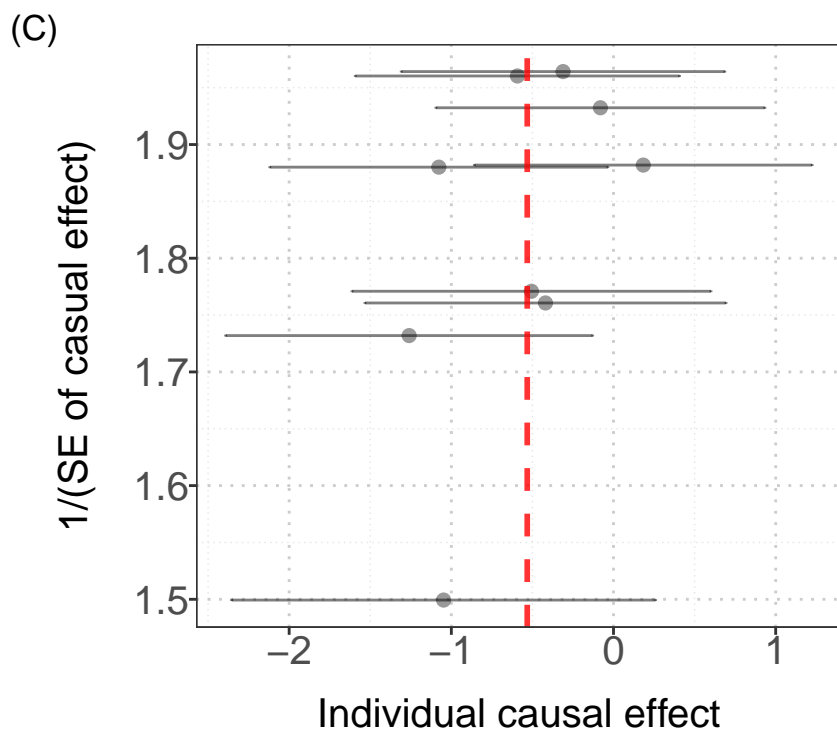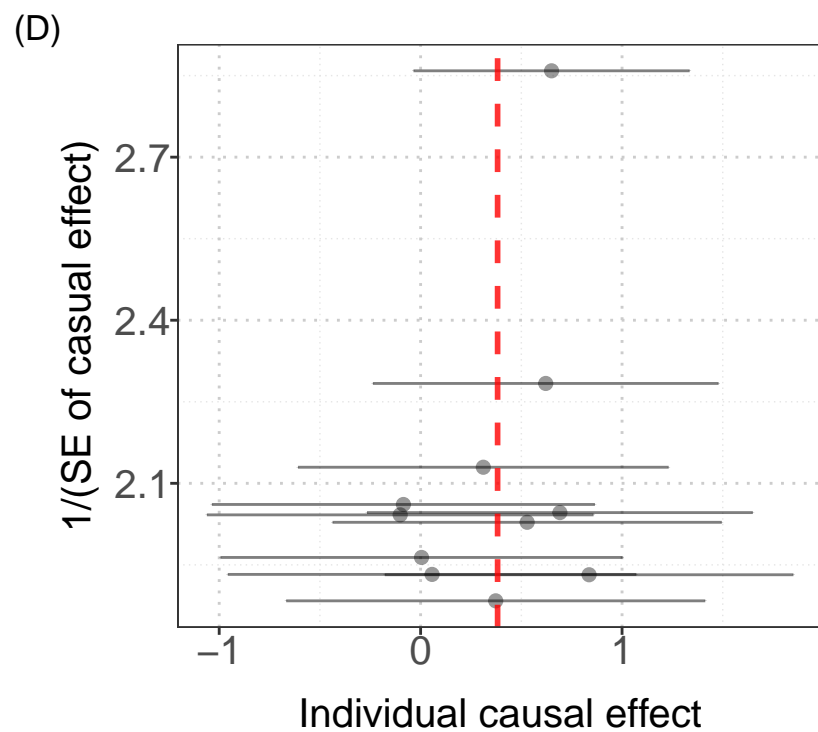

Supplement: Supplementary file 1 — Supplementary file1 (PDF 10 KB) [file 10142_2023_1054_MOESM1_ESM.pdf]

| Gut               | Pvalue | OR(95%CI)          |
|-------------------|--------|--------------------|
| Streptococcus     | 0.219  | 1.170(0.911–1.503) |
| Olsenella         | 0.195  | 1.107(0.949–1.290) |
| Enterobacteriales | 0.009  | 0.640(0.459–0.894) |
| Akkermansia       | 0.278  | 1.137(0.902–1.433) |

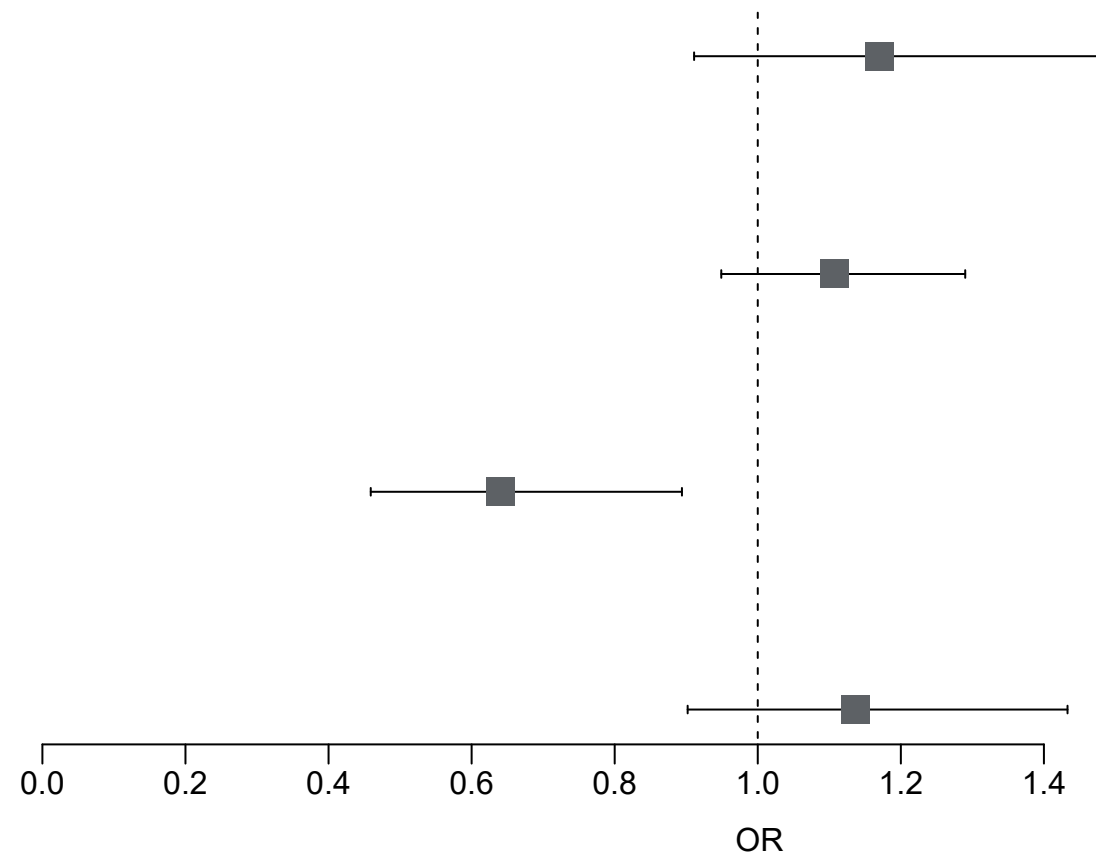

Supplement: Supplementary file 3 — Supplementary file3 (PDF 128 KB) [file 10142_2023_1054_MOESM3_ESM.pdf]
