# Supplementary material for: The causal role of intestinal microbiome in development of pre-eclampsia
Source: Funct Integr Genomics. 2023 Apr 17;23(2):127. doi: 10.1007/s10142-023-01054-8 (PMC10110674; doi:10.1007/s10142-023-01054-8)
Supplement: Supplementary file 2 — Supplementary file2 (PDF 5 KB) [file 10142_2023_1054_MOESM2_ESM.pdf]

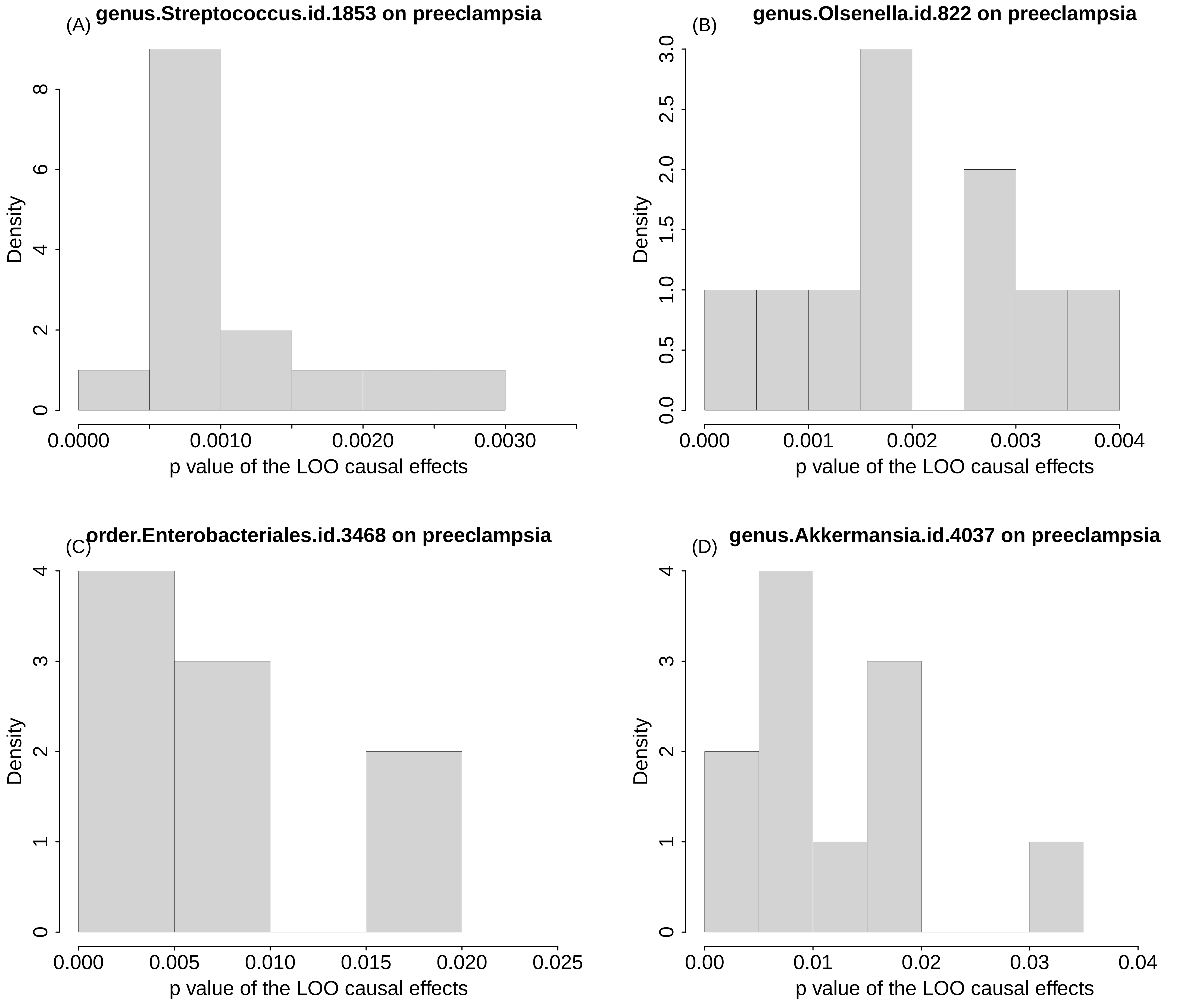
(A)
genus.Streptococcus.id.1853 on preeclampsia
Density
0
2
4
6
8
0.0000
0.0010
0.0020
0.0030
p value of the LOO causal effects
(B)
genus.Olsenella.id.822 on preeclampsia
Density
0.0
0.5
1.0
1.5
2.0
2.5
3.0
0.000
0.001
0.002
0.003
0.004
p value of the LOO causal effects
(C)
order.Enterobacteriales.id.3468 on preeclampsia
Density
0
1
2
3
4
0.000
0.005
0.010
0.015
0.020
0.025
p value of the LOO causal effects
(D)
genus.Akkermansia.id.4037 on preeclampsia
Density
0
1
2
3
4
0.00
0.01
0.02
0.03
0.04
p value of the LOO causal effects
